# Structured Mentorship Program for Women in Gastroenterology: Feasibility and Early Outcomes

**DOI:** 10.1016/j.gastha.2026.101010

**Published:** 2026-05-14

**Authors:** Leena Sai Penumalee, Sophie Brigstocke, Julia Vermylen, Wellness Working Group, Nirmala Gonsalves, Laya Charara, Bridale Handy-Robinson, Tiffani Darling, John Pandolfino, Ronak V. Patel

**Affiliations:** 1 Internal Medicine Residency Program, Department of Internal Medicine, Feinberg School of Medicine, Northwestern University, Chicago, Illinois; 2 Division of Gastroenterology and Hepatology, Feinberg School of Medicine, Northwestern University, Chicago, Illinois; 3 Division of Hospital Medicine, Feinberg School of Medicine, Northwestern University, Chicago, Illinois; 4 Office of Well-Being, Northwestern Medical Center, Chicago, Illinois

Women are underrepresented within gastroenterology (GI), comprising only 20% of attendings and 38% of fellows within the field.^1^ They also experience higher rates of burnout and imposter syndrome (IS).^2^^,^^3^ Mentorship positively impacts professional development and career satisfaction of women in academic medicine.^2^ Gender-concordant mentorship is also highly valued but difficult to obtain.^4^ There are few structured mentorship programs for women in GI and limited data on their effectiveness. We designed a novel mentorship program for female gastroenterologists as a quality improvement initiative and evaluated the program through structured preintervention and postintervention surveys.

All faculty members and trainees (ie, general and advanced fellows) who identified as female within the Division of Gastroenterology and Hepatology at a single large, tertiary care academic medical center were invited by email to participate in the mentorship program. The email included a link to an anonymous presurvey, which queried information about their professional role, perceptions of mentorship support, and topics they wanted to discuss during the program. Burnout and IS were also measured via the 2-item Maslach Burnout Inventory and the Young Imposter Scale, respectively.^5^^,^^6^ Completion of the survey signified consent. Participants were then divided into small groups of varied career stages by program leaders (S.B. and R.V.P.).

Mentorship groups met 3 times at 1-month intervals. Meeting themes were selected from salient topics identified by subjects in the presurvey; the 3 most prioritized topics included IS, career advancement, and work-life balance. Meetings were self-directed using discussion guides created and disseminated by authors (S.B., R.V.P.). Each guide included a brief introduction to the topic, summary of recently published literature, and 5–6 prompts asking participants to reflect on their experiences. Each participant was given a $50 gift card for purchasing food or beverages during the hour-long meetings. At the end of the study, all participants received an anonymous postsurvey in which they completed measures of burnout and IS, identified program strengths and weaknesses, and provided feedback. The study was reviewed and deemed exempt by the Northwestern University Institutional Review Board (IRB #00221003).

Invitations were sent to 23 female gastroenterologists (16 faculty members and 7 fellows) within a division of 49 faculty and 18 fellows (5 advanced fellows and 13 general fellows). The final study included 16 participants (11 faculty and 5 fellows, 70% participation). Participating faculty included 8 junior faculty (≤10 years of practice) and 3 senior faculty (>10 years of practice). Five mentorship groups were created, 4 groups with 3 participants each and 1 group with 4 participants. All groups met 3 times at 1-month intervals, except for 1 group which met twice due to scheduling difficulties. All participants completed both preintervention and postintervention surveys. Preintervention and postintervention measures were compared to assess intervention impact. Outcomes were analyzed descriptively due to small sample size.

Prior to the program, 38% of faculty members and trainees reported having access to strong mentors within the GI community. Only 19% reported having female mentors within the division; rates were higher among trainees (40%) than faculty (9%). After the program, 63% of participants (55% of faculty, 80% of trainees) reported improvement in their ability to connect with career and life mentors.

A score greater than 3 (scale 0–6) on either item of the 2-item Maslach Burnout Inventory was considered a positive finding of burnout. Thirty-eight percent of all participants screened positive for burnout at baseline. Postintervention, the overall rate of burnout was 31%. Faculty burnout rates decreased from 45% to 36%, but rates among trainees (20%) were unchanged (Figure).

**Figure:**
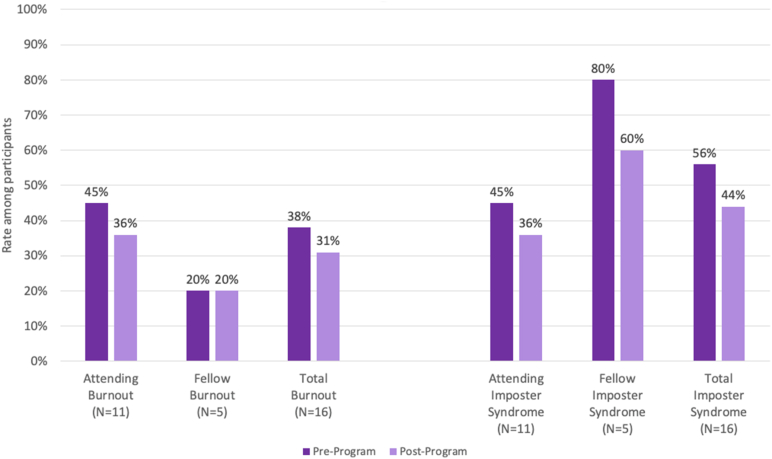
Comparison of preintervention and postintervention rates of burnout^1^ and imposter syndrome^2^ among participants (N = 16). ^1^Burnout was defined by a score >3 (scale 0–6) on either item of 2-item Maslach Burnout Inventory. ^2^Imposter syndrome was defined by answering “yes” to 5 or more questions on the Young Imposter Scale.

Answering “yes” to 5 or more of the questions on the Young Imposter Scale was considered a positive finding of IS. Nine participants (56%) screened positive for IS prior to the study, including 4 fellows (80%) and 5 faculty (45%). Seven participants (44%) screened positive for IS after the program, including 3 fellows (60%) and 4 faculty (36%) (Figure).

All (100%) participants found the program helpful to build community, and a majority (81%) reported an increased sense of belonging within the division. Eighty-one percent found the program helpful for developing professional and personal life skills. All (100%) participants wanted to continue engaging in the program and would recommend involvement to their female colleagues.

Two key findings from this study were the reported lack of mentorship (specifically gender-concordant mentorship) and notable rates of burnout and IS among participants. These findings highlight the need for interventions to improve professional growth for women in GI.

Women across all career stages reported difficulty in finding female mentors, and the skewed gender distribution in GI likely makes it more difficult for faculty members to find gender-concordant mentorship. Most participants reported improvement in their ability to connect with career and life mentors after the program. In addition to facilitating valuable connections among female gastroenterologists, mentorship programs normalize discussion on topics such as gender bias, micro-aggressions, fertility, and work-life integration.^7^ Group mentorship, as in this program, also fosters psychological safety and professional identity formation for trainees in GI. Having women in visible leadership roles also improves recruitment rates and cultivates an inclusive environment.^8^

Rates of burnout and IS among female faculty and trainees were high and consistent with previous literature.^3^^,^^9^ Strong connection with trusted mentors and robust institutional support are key protective factors against burnout and IS.^3^^,^^9^^,^^10^ There were small but encouraging decreases in rates of burnout and IS after the program. Expanding the program (ie, timeline, breadth of discussed topics) may allow for further improvements in these measures.

Program strengths included a manageable time commitment—highlighted by a 100% completion rate—and inclusion of individuals at various career stages. The program’s minimal administrative burden, low cost, and adaptability to virtual settings facilitate broad dissemination. Limitations include single-institution design and reliance on self-reported outcomes. Selection bias toward motivated participants and short follow-up duration limit generalizability and assessment of sustained impact. Future directions include developing more structured mentorship sessions, incorporating large group meetings, and scaling this mentorship framework to other GI departments or national societies.
